# Structure of an *Acinetobacter* Broad-Range Prophage Endolysin Reveals a C-Terminal α-Helix with the Proposed Role in Activity against Live Bacterial Cells

**DOI:** 10.3390/v10060309

**Published:** 2018-06-06

**Authors:** Nina N. Sykilinda, Alena Y. Nikolaeva, Mikhail M. Shneider, Dmitry V. Mishkin, Artem A. Patutin, Vladimir O. Popov, Konstantin M. Boyko, Natalia L. Klyachko, Konstantin A. Miroshnikov

**Affiliations:** 1 Shemyakin—Ovchinnikov Institute of Bioorganic Chemistry, Russian Academy of Sciences, Moscow 117997, Russia; sykilinda@mail.ru (N.N.S.); mm_shn@mail.ru (M.M.S.); 2 National Research Center “Kurchatov Institute”, Moscow 123182, Russia; nikolaeva_ay@nrcki.ru (A.Y.N.); vpopov@inbi.ras.ru (V.O.P.); kmb@inbi.ras.ru (K.M.B.); 3 Department of Chemistry, Lomonosov Moscow State University, Moscow 119991, Russia; dimami94@yandex.ru (D.V.M.); nlklyachko@gmail.com (N.L.K.); 4 Skryabin Moscow State Academy of Veterinary Medicine and Biotechnology, Moscow 109472, Russia; grenom96@yandex.ru; 5 Bach Institute of Biochemistry, Research Center of Biotechnology of the Russian Academy of Sciences, Moscow 119071, Russia

**Keywords:** endolysin, peptidoglycan hydrolase, *Acinetobacter*, prophage, structure, α-helix

## Abstract

Proteins that include enzymatic domain degrading the bacterial cell wall and a domain providing transport through the bacterial outer membrane are considered as prospective compounds to combat pathogenic Gram-negative bacteria. This paper presents an isolation and study of an enzyme of this class naturally encoded in the prophage region of *Acinetobacter baumannii* AB 5075 genome. Recombinant protein expressed in *E. coli* exhibits an antimicrobial activity with respect to live cultures of Gram-negative bacteria reducing the population of viable bacteria by 1.5–2 log colony forming units (CFU)/mL. However the protein becomes rapidly inactivated and enables the bacteria to restore the population. AcLys structure determined by X-ray crystallography reveals a predominantly α—helical fold similar to bacteriophage P22 lysozyme. The С-terminal part of AcLys polypeptide chains forms an α—helix enriched by Lys and Arg residues exposed outside of the protein globule. Presumably this type of structure of the C-terminal α—helix has evolved evolutionally enabling the endolysin to pass the inner membrane during the host lysis or, potentially, to penetrate the outer membrane of the Gram-negative bacteria.

## 1. Introduction

Peptidoglycan hydrolases, usually soluble bacteriophage endolysins and lytic enzymes encoded by bacterial genomes (autolysins) have attracted increased interest in the past decade, particularly in the context of emerging antibiotic resistance, and a desperate need in new potential antibacterials [1,2,3]. Bacteriophage endolysins are expressed on the late stage of the bacteriophage infection cycle, hydrolyzing the bacterial cell wall peptidoglycan. This leads to host cell osmotic lysis and phage progeny release. Bacterial metabolism also includes enzymes with similar function required for cell division and biofilm formation [4,5]. Starting from the classical work of Delbrück [6] demonstrating the use of these proteins against Gram-positive bacteria, a practical interest to use exogenous endolysins in medical and industrial applications exists. Lysins feature reasonable selectivity in targeting pathogenic speciesbut preserving commensal microflora [7]. Compared to acquired resistance against antibiotics, bacterial resistance against lysinsisvery low, as well as the potential of developing such a resistance [1,7,8]. Application of such enzymes, though, is very limited when spatial obstacles prevent direct enzyme contact with peptidoglycan. These shielding moieties may consist of outer membrane of Gram-negative bacteria or extracellular polysaccharides in case of biofilms and bacterial capsules. To permeabilize the outer membrane, so the lysins could reach their substrate in Gram-negative pathogens, EDTA or transmembrane peptides [9] are usually used. In the recent years a concept of “artilysins” has evolved. The term “Artilysin(R)” is a registered trademark owned by Lysando AG in the European Union, United States, and other countries. Artilysins are engineered chimeric proteins consisting of a catalytic part and a transmembrane domain providing penetration through outer membrane [10]. “Artilysation” by addition of such domain to known peptidoglycan hydrolases enhances their catalytic properties and ability to effect live bacterial cells without permeabilizing additives [11].

A number of reports about natural lysins exhibiting activity against both Gram-positive and Gram-negative bacteria were published (reviewed in [3]), but usually with no structural analysis of such proteins. Later, the genes encoding proteins with predicted bacteriolytic activity and transmembrane functions were found in many bacterial genomes, usually in their prophage regions. An activity of such recombinant proteins in vitroandin vivowas displayed [12], as well as the role of prophage endolysins in bacterial autolysis was demonstrated [13]. Therefore, a detailed study of “naturally artilyzed” peptidoglycan hydrolase may be beneficial for optimal design and antibacterial application of this enzyme class.

During an analysis of *Acinetobacter baumannii* strain AB 5075 genome we have identified an open reading frame (ORF) encoding a protein with the proposed muramidase activity that structurally resembles bacteriophage endolysins. This enzyme carries a C-terminal domain with predicted α-helical organization and a pronounced localized positive charge. An investigation of physico-chemical and structural properties of the recombinant version of this protein denoted as AcLys is the topic of the reported project.

## 2. MaterialsandMethods

### 2.1. Bacterial Strains

*Acinetobacter baumannii* strain AB 5075 (MRSN959) is originated from the Multidrug-Resistant Repository and Surveillance Network [14], and was a kind gift from PetrLeiman, University of Texas, Galveston. Bacterial strains used for lytic range determination were kindly provided by Olga S. Darbeeva, Tarasevich State Institute of Standardization and Control of Biomedical Preparations, Moscow, and are indicated in Table 1.

All bacterial strains used were stored frozen at −70 °C in 20% (*v*/*v*) glycerol. Frozen glycerol stocks were used to grow cells in LB broth at 37 °C.

### 2.2. MolecularCloning

Bacterial ORF encoding a putative AB 5075 prophage endolysin (AcLys) (WP_000208716.1) was PCR-amplified using primers 5′-TCTGTATTTCCAGGGATCCACAACTAAACCATTCTTCGATGC (Forward) and 5′-GTGCGGCCGCAAGCTTACTTCTTTAAAAAGAGTGCTC (Reverse), and cloned to the plasmid pEE3 using BamHI and HindIII restriction sites. Vector pEE3 was derived from pET23a (Merck, Germany) by introducing TEV protease (a Tobacco Etch Virus nuclear-inclusion-a endopeptidase) cleavage site immediately upstream of the BamHI restriction site. The resulting proteins contained the MGSSHHHHHHSSGQNLYFQGS tag at the N terminus (a His-tag followed by a TEV cleavage site). The gene encoding the N-terminally tagged protein was excised using NcoI and HindIII restriction endonucleases, and re-cloned to pBAD24 vector under control of the *ara* promotor.

### 2.3. Expression and Purification of the Recombinant Protein

*E. coli* strainDH10Bwas transformed with pAcLys plasmid. The cell culture was grown under intensive aeration at 37 °C in 2× YT liquid broth supplied with ampicillin (100 µg/mL). Protein synthesis was induced with the addition of arabinose to a final concentration of 0.2% (*w*/*w*) when the culture density reached OD_600_ ~ 0.3, and incubation continued for another 3 h. Cells were harvested by centrifugation at 3000× *g* for 20 min at 4 °C (rotor Beckman JA-14, Brea, CA, USA). The cell pellet was resuspended in 20 mM Tris pH 8.0, 200 mM NaCl (1/50 volume of the cell culture), and cells were disrupted by sonication on wet ice (VirSonic 100, VirTis, Manasquan, NJ, USA). Cell debris was removed by centrifugation (12,000× *g*, 20 min, 4 °C, rotor Beckman JA-17) and the supernatant filtered by passage through a 0.45 µm filter (Millipore Corporation, Bedford, MA, USA). Then supernatant was applied to Ni-Chelating Sepharose Fast flow column (GE Healthcare, Little Chalfont, UK) and chromatographically purified by linear 0–200 mM imidazole gradient in 20 mM Tris pH 8.0, 200 mM NaCl using a FPLC chromatography system (Pharmacia) [15]. Fractions revealing enzyme activity were pooled, β-mercaptoethanol was added to 1 mM concentration, and the 6× His-tag was removed using 10 µg/mL TEV protease by overnight incubation at 4 °C. The reaction mixture was reapplied to Ni-chelating column, and the flow-through fractions were concentrated using Amicon Ultra-4 Centrifugal Filters Ultracel-10K (MILLIPORE IRELAND Ltd). AcLys protein was additionally purified by gel filtration using Superose 12 HR10/30 column (GE Healthcare) equilibrated with 50 mM Bis-Tris pH 6.0. Samples were checked for purity using 12% SDS-polyacrylamide gel electrophoresis [16]. Protein concentration was determined using a Pierce BCA Protein Assay Kit (Thermo Scientific, Waltham, MA, USA). AcLys enzyme of normalized concentration was supplied with 0.04% sodium azide, 0.1 mM PMSF (phenylmethylsulfonyl fluoride), 10% glycerol, and stored at −70 °C.

### 2.4. Substrate Specificity Assay

Kinetics of AcLys influence on viable bacterial cells was determined turbidimetrically using a 96-well plate reader Bioscreen C (Thermo Scientific, LabsystemsOy). Different concentrations of AcLys (1, 10, 50, 100, 200, 400 µg/mL)were dosed at time zero into 200 µL of microbial suspensionsin LB media collected at mid-log growth phase (OD_600_ = 0.4) andnormalized to approximately 5 × 10^8^ CFU/mL with 20 mM Bis-Tris (pH 6.0). Optical density was monitored for 30 min with periodic shaking at room temperature. To reveal the influence of the incubation conditions on bacteriolytic activity the collected cells were centrifuged at 1500× *g*, washed with deionized water, and resuspended to the corresponding optical density with 20 mM Bis-Tris (pH 6.0). To calculate the minimum inhibitory concentration (MIC) the incubation of the reaction mixture was continued further, and the number of live cells was determined by removing aliquots from the microbial suspension at 15, 30, 60, 120, 240 min of incubation with the enzyme, seriallydiluting themand platingdilutions on nutrient agar. All measurements were performed in triplicates. The MIC of AcLys was defined as a concentration of an enzyme that reduces the bacterial population at least 10-fold, and inhibits the growth of the bacteria within a 2 h interval.

### 2.5. Activity Assay

Peptidoglycan degrading activity was quantitatively assayed by the turbidimetric methodmainlyas described in [17]. A 96-well plate/format was used for preliminary screening, and Ultrospec 3000 (Pharmacia) spectrophotometer with 1 cm cuvettes was used for precise measurements. The OD was monitored at 600 nm. All assays at all conditions were performed in triplicate. *E. coli* CR63 cells were harvested by centrifugation, washed with deionized water, diluted to OD_600_ = 0.6 with 20 mM Bis-Tris buffer pH 6.0, aliquoted and stored at −70 °C. Routinely the assays were recorded in 20 mM Bis-Tris (pH 6.0) at 25 °C using 1 µg/mL AcLys concentration. Temperature dependence of the enzyme activity was assessed by thermostating the reaction mixture at corresponding temperatures. To study the effect of ionic strength and inorganic cations, various concentrations of NaCl, KCl, CaCl_2_ or MgCl_2_ were added to the reaction mixture. To estimate the influence of pH on activity, the permeabilized cells were resuspended in 20 mM Tris-HCl/20 mM citrate/20 mM phosphate buffer with pH adjusted with NaOH in the range of 4.5–9. Then the enzyme was added, and the decrease of optical density was monitored. Thermal inactivation of AcLys was studied adding the enzyme from the stock incubated at stated temperature for a certain period of time, and the reaction was run at 25 °C. Enzymatic activity was calculated as the steepest slope of OD_600_ versus time curve expressed in ΔOD_600_/min units.

### 2.6. Protein Crystallization, Data Collection and Processing, Structure Solution

AcLys was crystallized using vapor diffusion technique. Microgravity conditions were produced on module Luch of International Space Station (expeditions ISS-51 and ISS-53) to decrease convection [18]and obtain high quality protein crystals. Crystals were grown at 20 °C in the following conditions: 0.2 M Li_2_SO_4_; 0.1 M MES (2-(*N*-morpholino)ethanesulfonic acid), pH 6.5; 25% PEG3350.

Crystals were briefly soaked in 20% (*v*/*v*) glycerol for cryoprotection. The X-ray dataset was collected at 100K to a resolution of 1.2 Å using Pilatus 6M-F detector on the BL41XU beamline at the SPring-8 synchrotron-radiation facility (Japan). The following data collection strategy was predicted by HKL2000 [19]: wavelength—0.8Å, rotation angle—140°, oscillation angle—1.0°, crystal to detector distance—210 mm. Data was processed with Mosflm [20]. Data collection statistics were summarized in Table 2.

Structure solution was made by molecular replacement method with BALBES program [21] using known structure of P22 lysozyme (PDB code 2ANV) as a starting model.

Structure refinement was made with Refmac5 [22] and COOT [23] programs. There is one protein monomer is the asymmetric unit. Resolution was gradually increased to 1.2 Å during the refinement. Hydrogens in rigid positions as well as anisotropic B-factor refinement were used on last stages of the refinement. The final model comprises one subunit of the protein (149 residues), 229 water molecules as well as sulfate and glycerol molecules. The first 47 N-terminal residues were not observed in electron density maps apparently due to high mobility of these residues or to limited proteolysis. The first suggestion is preferred because there are indistinguishable electron density peaks near the protein surface which could be fragments of disordered N-termini. All stereochemical parameters for side-chain and main-chain atoms were within acceptable limits, with the φ–ψ values of the residues being in the most favored (98%) or allowed (2%) regions of the Ramachandran plots. Structure was verificated with Molprobity [24] and PDB_REDO [25] servers. Final refinement statistics is summarized in Table 2. The visual inspection of the structure model was carried out with the COOT and Pymol (The PyMOL Molecular Graphics System, Version 1.2r3pre, Schrödinger, LLC). Comparison of the structures was made with the PDBeFOLD program [26]. The contacts were analyzed using the PDBePISA [27]. Structural data were deposed to the Protein Data Bank (www.rcsb.org) with the accession code 6ET6.

## 3. Results

### 3.1. Bioinformatic Analysis

The genome of AB 5075 strain of *Acinetobacter baumannii (*NCBI accession number GCA_000241685.2) contains a prophage region. If inducible, this prophage should form a Siphovirus similar to Βϕ-R3177 [28]. The lysis cassette of this phage contains ORFs encoding putative holin (ABUW_RS06465), Rz-like protein (ABUW_RS06460) and an endolysin (ABUW_RS06470). The latter protein denoted as AcLys has a predicted peptidoglycan-lysing activity. Conservative domain (aa 40–177) is typical for phage endolysins and bacterial autolysins, and usually degrades peptidoglycan by a *N*-acetyl-β-d-muramidase activity utilizing a Glu-Asp-Thr catalytic triad [29]. In the case of AcLysresidues catalytic roles are presumably played by Glu 52 (proton donor), Asp 61 (nucleophile), and Thr 67 (stabilizer). Among well studied peptidoglycan hydrolases with conserved active site residues the sequence of AcLys has the similarity most close to the lysozyme of *Salmonella* phage P22 (47% amino acid identity, E value 2 × 10^−35^) [30]. Genes encoding sequences similar to AcLys (above 80% identity) can be found by BLAST [31] within the genomes of many sequenced strains of *Acinetobacter*genus, and are usually attributed as prophage lysozymes. Unlike endolysins of phages infecting Gram-positive bacteria that possess separate catalytic and cell-wall binding domains, Gram-negative-specialized endolysins usually contain a single globular catalytic domain, with but a few exceptions. So are AcLys and similar putative endolysins of *Acinetobacter* prophages. The C-terminal part of AcLys polypeptide contains a long trail of Arg and Lys residues. HHPred analysis [32] predicted this part of the polypeptide chain to be organized as an α-helix. Such positively charged peptide may contribute to the enhanced ability to penetrate the outer membrane and reach the peptidoglycan substrate, and this fact stimulated the selection of this protein for further study.

### 3.2. Molecular Cloning and the Properties of Recombinant AcLys

Recombinant AcLys demonstrated pronounced cytotoxicity with respect to *E. coli* cells B834 (DE3) when expressed with IPTG induction (T7 expression system). Therefore, the tightly regulated arabinose promotor was used for recombinant synthesis of AcLys. Optimized expression conditions and a combination of metal affinity and gel permeation chromatography yielded ~40 mg of active protein of >95% purity from 1 L cell culture. The removal of the N-terminal 6× His affinity tag results in insufficient increase of activity and no change in protein solubility or stability. Gel permeation chromatography of AcLys shows that the protein exists as a dynamic mixture of monomer and dimer in solution. Both monomer and dimer fractions demonstrate identical mobility in SDS-PAGE. Electrophoretic mobility of AcLys corresponds to the calculated Mw of 21.6 kDa. The calculated extinction coefficient of 19,940 M^−1^cm^−1^ at 280 nm was further used to assess the concentration of the purified protein.

### 3.3. Substrate Specificity Assay

**Figure 1:**
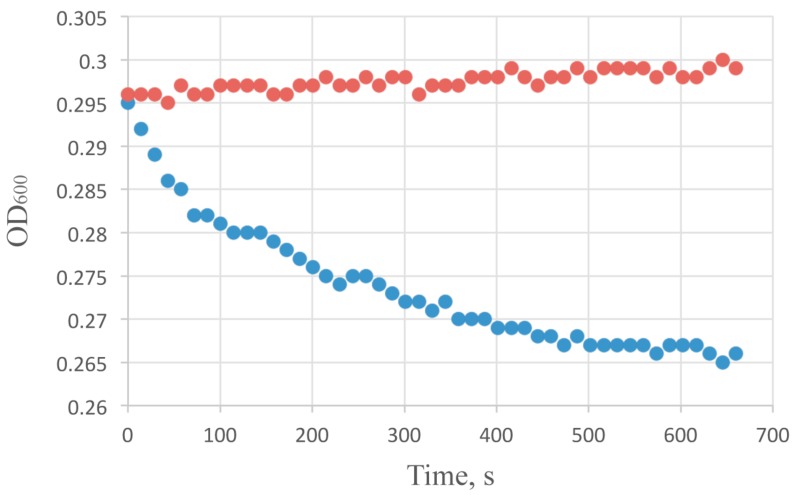
Bactericidal activity of AcLys (100 µg/mL) on viable *E. coli* CR63 cells in LB media normalized to pH 6.0 with Bis-Tris buffer at 25 °C (blue). Background *E. coli* CR63 optical density at the same conditions with no enzyme added is shown in red.

The results of substrate specificity measurements using a panel of 10 bacterial strains, representing 6 species of Gram-negative and 2 of Gram-positive bacteria, are presented in Table 1. The short incubation time have resulted in fast but rather small reduction of optical density (Typical optical density curve is presented at Figure 1). The count of viable cells in the reaction mixture has dropped significantly during AcLys application, about 1.5–2 log within 15–30 min for *E. coli* and *P. aeruginosa*. Several points were found to be essential to design further experiments: (i) An effect of AcLys on *E. coli*, *P. aeruginosa* and *K. pneumoniae* cells was about 2-fold more pronounced compared to the strains of *Acinetobacter*. This effect can be explained by similar substrate specificity of enzyme towards Gram-negative peptidoglycan, and was observed for a number of endolysins originated from phages infecting *Acinetobacter* [33,34] Gram-positive bacteria were not affected by AcLys at comparable concentrations; (ii) After ~30 min of incubation the bacteriolytic effect of AcLys has terminated, and after 1–2 h lag period (dependent on applied enzyme concentration) the bacterial density started to grow gradually. So if the MIC is conventionally calculated with respect to the overnight incubation of the bacterial culture, as it is used for the estimation of antibiotic efficacy, then the effect of AcLys should be considered bacteriostatic or even unobservable on a longer timescale. The action of this enzyme tends to be limited in time, presumably because of its fast inactivation and/or irreversible binding to the bacterial surface. So we set the MIC as the minimal enzyme concentration that causes at least 10× decrease in cell counts and prevents further cell growth for 2 h in the conditions of bacterial cultivation; (iii) Sedimentation of the cells and washing them with deionized water before normalizing the bacterial concentration for activity assay caused more defined antibacterial effect (>3 log population drop vs. 1.5–2 log when the assay was processed in the LB media) (Figure S1). So the reaction conditions play an essential role for enzyme activity, where cells become partially permeabilized by hypotonic conditions, and the inhibitory role of the ionic strength is minimized (see further). The similar effect when the conditions close to pure water were optimal for an enzyme activity were observed for other recombinant lysins from *Acinetobacter* phages [33,35]; Therefore further assays to assess the enzymologic properties of AcLys were performed using the suspension of *E. coli* cells partially permeabilized by freezing in storage and with growing media components removed by washing with water. In such conditions even the minimal concentrations of the enzyme (below 1 µg/mL) were sufficient to monitor the reduction of optical density.

### 3.4. Activityand Optimal Enzymologic Conditions of AcLys

**Figure 2:**
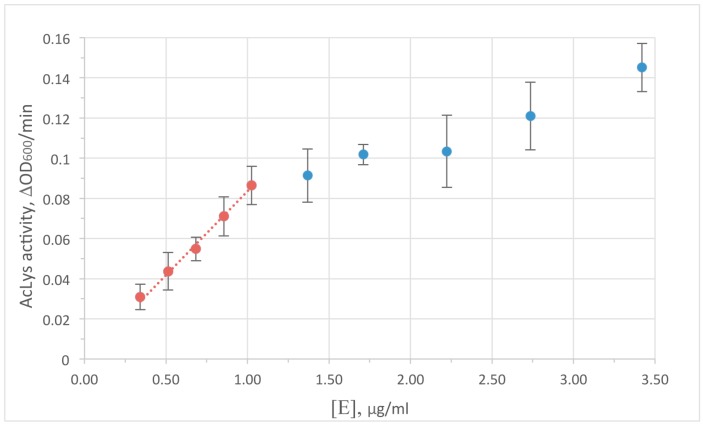
Dependence of AcLys activity on the enzyme concentration in the reaction mixture. An initial density of bacterial cell suspension OD_600_ = 0.6. The substrate is *E. coli* CR63 preliminary washed with deionized water and frozen at −70 °С. Reaction conditions 20 mM Bis-Tris (pH 6.0), 25 °C.

Turbidimetric method was used to monitor the enzyme activity. A decrease in optical density in the cell suspension is caused by the degradation of peptidoglycan bonds with subsequent osmotic cell lysis, and yields a typical saturation curve. Peptidoglycan hydrolysis is the limiting step of the reaction at low enzyme concentrations. So the concentrations range 0–1 μg/mL AcLysin the reaction mixture was chosen to satisfy requirements for the early linear range of the curve for further experiments (shown at Figure 2). It is worth to note that the concentration of an enzyme resulting in the similar reduction of optical density for accessible peptidoglycan (permeabilized cells) and for live bacterial cells protected by outer membrane differs by nearly 2 orders of magnitude (1 vs. 100 μg/mL). This difference is substantially smaller than usually observed for most reported single-domain globular lysins to cause antibacterial effect in the live cells without additives. Therefore, an ability of AcLys to pass through the outer cell membrane autonomously can be considered as a feature of this protein.

**Figure 3:**
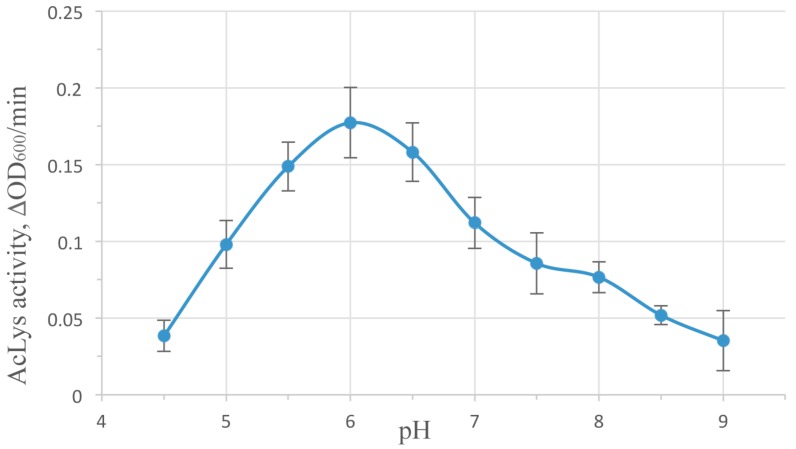
pH influence on AcLys activity. The reactions were run in 10 mM Tris-HCl, 10mM K_2_HPO_4,_ 10 mM Na acetate adjusted to corresponding pH with NaOH at 25 °С. Enzyme concentration 1 µg/mL. Substrate—*E. coli* CR63, preliminary washed with deionized water and frozen at −70 °С.

AcLys demonstrates high enzymatic activity in the pH range of 4.5–9 peaking at pH 6 (Figure 3). This value is typical for most peptidoglycan-degrading enzymes of phage origin, though it differs from pH 8.0 shown as optimal for very similar phage P22 lysozyme [36]. Temperature in ambient limits (25–40 °С) plays a few role in the enzyme activity, with a slight maximum at 30 °С (Figure S2). Ionic strength influences the lytic activity of AcLys significantly (Figure S3). Generally as salt concentration in the buffer solution rises, the activity decreases. Divalent cations Ca^2+^, Mg^2+^ exhibit more effect (80% inhibition at 2 mM concentration) compared to monovalent Na^+^, K^+^ (80% inhibition at 80mM). Reverse effect activity vs. salt concentrations is observed for many known peptidoglycan hydrolases [33,35], including phage P22 lysozyme [36], but is never described as much pronounced.

### 3.5. Oligomeric State of AcLys

Size-exclusion chromatography of recombinant AcLys shows an equilibrium of two states of the protein in solution. After 3–4 h after separation of monomers and dimers the components of each fraction redistribute back to the mixture of monomer:dimer. The feature of the dynamic equilibrium of oligomeric states of the protein in solution was observed previously, for instance, for endolysin gp144 of *Pseudomonas* bacteriophage ϕKZ [37]. The share of monomeric form is higher when the protein solution is dilute. When freshly isolated, the enzymatic activity of the monomeric form is ~3-fold higher than that of the dimer. The reason may be the limited accessibility of the peptidoglycan substrate to the active site of the dimeric form or spatial troubles with substrate accessibility to dimeric form of protein. Dynamic dimerization of protein globule was also observed during protein crystallization (see further). AcLys polypeptide chain contains the only Cys99 residue. An addition of β-mercaptoethanol does not affect dimerization and overall protein stability. Therefore, the dimer formation does not involve covalent disulfide bridging.

### 3.6. Stability of AcLys Protein

**Figure 4:**
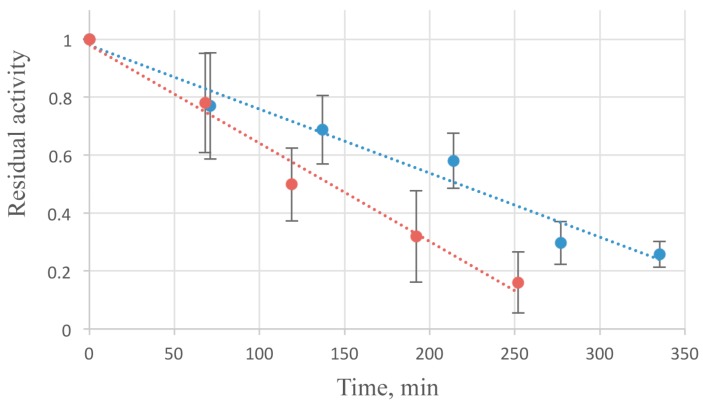
AcLys thermal inactivation kinetics at 37 °C. The substrate *E. coli* CR63 were washed with deionized water and frozen at −70 °С. AcLys stock in 20 mM Bis-Tris (pH 6.0) with the addition of 10 mM NaCl (red), or 300 mM NaCl (blue) was incubated at 37 °C for indicated time periods, then the enzyme was added to the standard reaction mixture to 1 µg/mL concentration, and the reaction ran at 25 °C.

All studiedthermalconditionsatpH 6.0 (4, 25, 37 and 50 °C) caused an irreversible thermal inactivation of the enzyme. About a half of enzymatic activity is lost in 2 h at 37 °C (Figure 4). Some stabilizing effect was observed while the addition of NaCl in 50–300 mM concentration.

An increase of ionic strength gradually enhanced the lifetime of the enzyme. It may be caused by the enhancement of the hydrophobic interactions inside the protein globule. Freezing of the enzyme after addition of 20% glycerol enables the long-term storage of AcLys. After 3 month incubation at −70 °С the activity have dropped by less than 10%.

### 3.7. Structural Analysis

**Figure 5:**
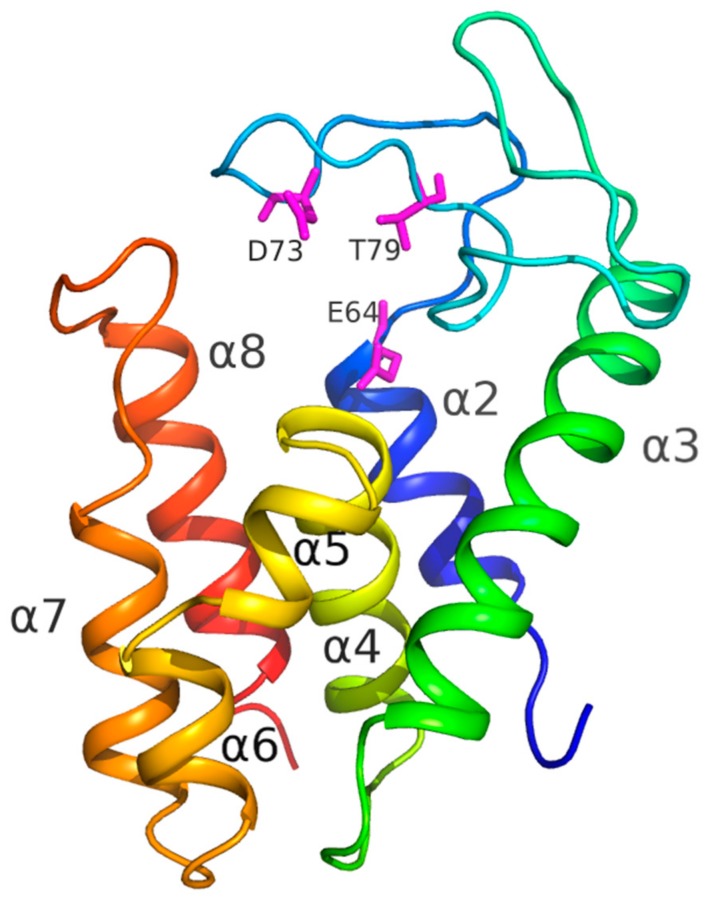
AcLys monomer. Molecule is colored in rainbow manner from blue on N-termini to red on C-termini. Secondary structure elements are captioned as well as the active site residues (pink).

The crystal structure of AcLys was refined to 1.2 Å resolution. The protein has overall α-helical architecture typical for lysozyme family [38], and is composed of seven α-helices (Figure 5): α2: residues 54–64, α3: 101–122, α4: 129–142, α5: 144–149, α6: 151–157, α7: 161–170 and α8: 180–193. Active site is formed by conserved catalytic triad: Glu64, Asp73 and Thr79 (Figure 5) are clearly seen in the electron density. Glu64 forms salt bridge to conserved Arg185 which lies on the C-terminal helix α8. This salt bridge is a well-known feature of the known endolysins of P21 and P22 families [38].

### 3.8. Comparison to Known Structures

**Figure 6:**
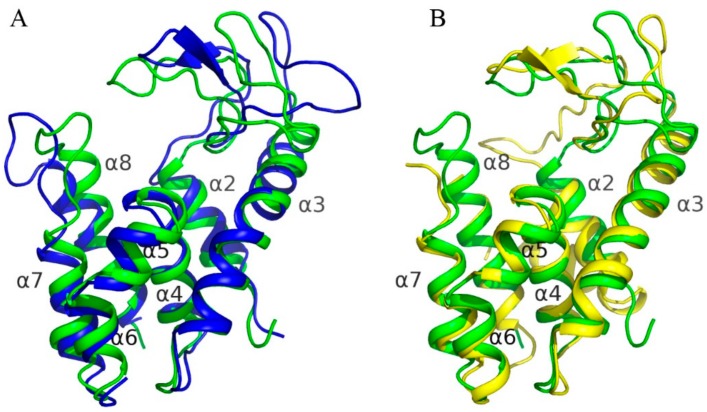
Superposition of AcLys to structures of homologue enzymes. AcLys is colored in green. Secondary structure elements were captioned in accordance to AcLys. (**A**) Superposition on P22 lysozyme (2ANV); (**B**) Superposition on full-length endolysin R21 from phage 21 (3HDE).

Superposition of AcLys structure on homologous structures revealed that all the structures are quite similar (Figure 6) with RMSD between corresponding Cα-atoms varying from 1.8 to 2.1 Å^2^. The major differences were found in looped regions connecting secondary structure elements, especially in long loops which connect α2–α3 and α7–α8 (Figure 6). At thesame time all α-helices stay near the same positions relative to homologous structures. Looped region connecting α2–α3 helices harbors a catalytic triad (Figure 5) and thus the difference in orientation of theses loops in the enzymes leads to a difference in size of their active site cavity. The major difference was found in position of catalytic Glu35 of endolysin R21 from phage 21 (PDB code 3HDE) which is located far away from position of analogous glutamic acid in homologous enzymes, including AcLys, due to backbone distortion.

In structures offull-length endolysin R21 from phage 21 and DLP12 endolysin from *E. coli* (4ZPU) there is an extra N-terminal helix—α1, which is absent in case of AcLys, T4 lysozyme (5JWS) as well as P22 lysozyme (2ANV). This helix together with helix α2 are known as a special SAR domain of endolysin R21 which is required to anchor the enzyme to a membrane [38].

С-terminal α8 helix of AcLysis supposed to play an important role during cell lysis, penetrating into cell membrane. Comparison to other enzymes with high sequence similarity did not reveal significant differences in the orientation of this helix, despite some displacement in its beginning caused by different orientation of loops between α7 and α8. In case of AcLys the loop harbors an increased number of positively charged residues, including conserved Arg185, Arg186, Arg190 and Lys180 (also found in case of DLP12 endolysin from *E. coli*), unique Arg184—which is a part of positively charged patch consisting of three arginines, as well as Lys195 and Lys196. These residues surround the loop forming positive charge on their surface and thus could potentially bind and penetrate the cell membrane.

## 4. Discussion

The presence of the specialized cell-wall binding domain in bacteriophage peptidoglycan hydrolases aimed to degrade the murein of Gram-positive bacteria is a common feature of such proteins. The local attachment of the lysin to the substrate enables it to concentrate its activity andprovides additional selectivity of the enzyme. The irreversible binding prevents lysis of the other bacterial cells in the course of the bacteriophage infection [1,2,3]. The role of the additional domain providing the binding to the cell surface is not obvious in the case of endolysins synthesized by bacteriophages infecting Gram-negative bacteria, and the number of known endolysins with such domains is small. The chemical composition of Gram-negative peptidoglycan is not that diverse, especially in its glycan part [39]. Therefore, most single-domain globular endolysins of Gram-negative phages demonstrate reasonable specificity. An access to the peptidoglycan of the adjacent cells in the bacterial population is impaired by the outer membranes. The possibility to modify endolysins to enable them attack Gram-negative bacteria from outside is a long term research interest in the context of therapeutic antibacterial application of such proteins. One of the strategies to overcome the outer membrane of the bacteria is the use of transmembrane peptides—either as a component of the reaction mixture [40] or as a fused part of the chimeric protein, “artilysin” [9,10,11]. One of the problems in artilysin design is the structural positioning of the engineered domain in the protein globule. We have identified that a number of putative proteins of phage and prophage origin possess terminal domains resembling transmembrane peptides, probably evolved naturally. AcLys, one of such “naturally artilyzed” protein with the C-terminal α-helical domain demonstrates moderate activity against viable Gram-negative bacteria from outside. X-ray structure of AcLys shows that the α-helical domain does not obstruct the structural integrity of the enzyme globule. Recombinant enzyme rapidly inactivates even in the absence of the substrate and its optimal functional conditions are far from being physiological, so the natural role of such enzyme in the process of the bacteriophage infection is unclear, as well as its applied antimicrobial potential. The exact function of the C-terminal α-helix in outer membrane binding and transmembrane transport is also a subject for future investigation. However, the difference between AcLys activity against permeabilized and live bacteria is not that striking as it is usually observed for Gram-negative endolysins, so it is possible to propose an active role of the C-terminal α-helical domain in the “lysis from without” process. The search of putative endolysins with pronounced α-helical domain within available genomic databases [41] may yield more active and stable recombinant protein. Alternatively, the “artilyzation” of the single-domain endolysin with required physico-chemical properties and specificity by fusing an α-helical domain with known sequence and structure can enhance the penetration of the resulting chimeric protein through the outer membrane of the cell. In this case the spatial positioning of the putative anchoring α-helix with respect to the protein globule should be taken into consideration.

## Figures and Tables

**Table 1:** AcLys substrate specificity assay.

Bacterium	Strain	Apparent MIC *, 2 h in LB, µg/mL
*Acinetobacter baumannii*	ATCC19606	100
*Acinetobacter baumannii*	AB5075	100
*Acinetobacter pitii*	ATCC17978	100
*Escherichia coli*	CF1073	50
*Escherichia coli*	CR63	50
*Klebsiella pneumoniae*	GISK 233	50
*Pseudomonas aeruginosa*	PAO1	50
*Proteus vulgaris*	GISK 185	100
*Bacillus thuringiensis*	GISK 202-E	-
*Staphylococcus aureus*	GISK 222	-

*: minimal inhibitory concentration; -: there was no inhibition.

**Table 2:** Data collection and refinement statistics of AcLys.

Data Collection		Refinement	
Space group	P22_1_2_1_	R_work_/R_free_	14.7/16.9
Cell dimensions		No. of atoms	
*a*, *b*, *c* (Å)	30.98; 67.00; 75.76	Protein	1152
α, β, γ (°)	90; 90; 90	Ligands/ion	11
Resolution (Å)	50.19–1.20 (1.22–1.20)	Water	229
R_meas_ (%)	12.4 (100.0)	Ramachandran outliers, %	0
CC_1/2_	99.4 (77.7)	Ramachandran favored, %	98
<I>/<σ(I)>	5.6 (1.5)	R.m.s deviations	
Completeness	94.0 (94.5)	Bond length (Å)	0.019
Redundancy	5.0 (5.0)	Bond angle (°)	0.980
		MolProbity Score	1.44

## Supplementary Materials

The following are available online at http://www.mdpi.com/1999-4915/10/6/309/s1, Figure S1: Removal of the growth media components enhances the effect of Aclys on live bacterial cells; Figure S2: Temperature dependence of AcLys activity; Figure S3: Dependence of AcLys activity on salt concentration.

## References

[1] Fischetti V.A. (2008). Bacteriophage lysins as effective antibacterials. Curr. Opin. Microbiol..

[2] Borysowski J., Weber-Dąbrowska B., Górski A. (2006). Bacteriophage Endolysins as a Novel Class of Antibacterial Agents. Exp. Biol. Med..

[3] Nelson D.C., Schmelcher M., Rodriguez-Rubio L., Klumpp J., Pritchard D.G., Dong S., Donovan D.M. (2012). Endolysins as Antimicrobials. Adv. Virus Res..

[4] Frirdich E., Gaynor E.C. (2013). Peptidoglycan hydrolases, bacterial shape, and pathogenesis. Curr. Opin. Microbiol..

[5] Vollmer W., Joris B., Charlier P., Foster S. (2008). Bacterial peptidoglycan (murein) hydrolases. FEMS Microbiol. Rev..

[6] Delbruck M. (1940). The growth of bacteriophage and lysis of the host. J. Gen. Physiol..

[7] Schuch R., Nelson D., Fischetti V.A. (2002). A bacteriolytic agent that detects and kills *Bacillus anthracis*. Nature.

[8] Schmitz J.E., Schuch R., Fischetti V.A. (2010). Identifying active phage lysins through functional viral metagenomics. Appl. Environ. Microbiol..

[9] Briers Y., Lavigne R. (2015). Breaking barriers: Expansion of the use of endolysins as novel antibacterials against Gram-negative bacteria. Future Microbiol..

[10] Gerstmans H., Rodriguez-Rubio L., Lavigne R., Briers Y. (2016). From endolysins to Artilysin(R)s: Novel enzyme-based approaches to kill drug-resistant bacteria. Biochem. Soc. Trans..

[11] Rodríguez-Rubio L., Chang W.-L., Gutiérrez D., Lavigne R., Martínez B., Rodríguez A., Govers S.K., Aertsen A., Hirl C., Biebl M. (2016). ‘Artilysation’ of endolysin λSa2lys strongly improves its enzymatic and antibacterial activity against streptococci. Sci. Rep..

[12] Gervasi T., Horn N., Wegmann U., Dugo G., Narbad A., Mayer M.J. (2014). Expression and delivery of an endolysin to combat *Clostridium perfringens*. Appl. Microbiol. Biotechnol..

[13] Visweswaran G.R.R., Kurek D., Szeliga M., Pastrana F.R., Kuipers O.P., Kok J., Buist G. (2017). Expression of prophage-encoded endolysins contributes to autolysis of *Lactococcus lactis*. Appl. Microbiol. Biotechnol..

[14] Zurawski D.V., Thompson M.G., McQueary C.N., Matalka M.N., Sahl J.W., Craft D.W., Rasko D.A. (2012). Genome sequences of four divergent multidrug-resistant *Acinetobacter baumannii* strains isolated from patients with sepsis or osteomyelitis. J. Bacteriol..

[15] Crowe J., Döbeli H., Gentz R., Hochuli E., Stüber D., Henco K. (1994). 6× His-Ni-NTA Chromatography as a Superior Technique in Recombinant Protein Expressiod/Purification. Protocols for Gene Analysis.

[16] Brunelle J.L., Green R. (2014). One-dimensional SDS-polyacrylamide gel electrophoresis (1D SDS-PAGE). Methods Enzymol..

[17] Briers Y., Lavigne R., Volckaert G., Hertveldt K. (2007). A standardized approach for accurate quantification of murein hydrolase activity in high-throughput assays. J. Biochem. Biophys. Methods.

[18] Boyko K.M., Popov V.O., Kovalchuk M.V. (2015). Promising approaches to crystallization of macromolecules suppressing the convective mass transport to the growing crystal. Russ. Chem. Rev..

[19] Otwinowski Z., Minor W. (1997). Processing of X-ray diffraction data collected in oscillation mode. Methods Enzymol..

[20] Battye T.G.G., Kontogiannis L., Johnson O., Powell H.R., Leslie A.G.W. (2011). iMOSFLM: A new graphical interface for diffraction-image processing with MOSFLM. Acta Crystallogr. Sect. D Biol. Crystallogr..

[21] Long F., Vagin A.A., Young P., Murshudov G.N. (2007). BALBES: A molecular-replacement pipeline. Acta Crystallogr. Sect. D Biol. Crystallogr..

[22] Vagin A.A., Steiner R.A., Lebedev A.A., Potterton L., McNicholas S., Long F., Murshudov G.N. (2004). REFMAC5 dictionary: Organization of prior chemical knowledge and guidelines for its use. Acta Crystallogr. Sect. D Biol. Crystallogr..

[23] Emsley P., Lohkamp B., Scott W.G., Cowtan K. (2010). Features and development of Coot. Acta Crystallogr. Sect. D Biol. Crystallogr..

[24] Chen V.B., Arendall W.B., Headd J.J., Keedy D.A., Immormino R.M., Kapral G.J., Murray L.W., Richardson J.S., Richardson D.C. (2010). MolProbity: All-atom structure validation for macromolecular crystallography. Acta Crystallogr. Sect. D Biol. Crystallogr..

[25] Joosten R.P., Long F., Murshudov G.N., Perrakis A. (2014). The PDB-REDO server for macromolecular structure model optimization. IUCrJ.

[26] Krissinel E., Henrick K. (2004). Secondary-structure matching (SSM), a new tool for fast protein structure alignment in three dimensions. Acta Crystallogr. Sect. D Biol. Crystallogr..

[27] Krissinel E., Henrick K. (2007). Inference of Macromolecular Assemblies from Crystalline State. J. Mol. Biol..

[28] Jeon J., D’Souza R., Pinto N., Ryu C.-M., Park J., Yong D., Lee K. (2015). Complete genome sequence of the siphoviral bacteriophage Βϕ-R3177, which lyses an OXA-66-producing carbapenem-resistant Acinetobacter baumannii isolate. Arch. Virol..

[29] Hardy L.W., Poteete A.R. (1991). Reexamination of the Role of Asp20 in Catalysis by Bacteriophage T4 Lysozyme. Biochemistry.

[30] Rennell D., Poteete A.R. (1989). Genetic analysis of bacteriophage P22 lysozyme structure. Genetics.

[31] Altschul S.F., Madden T.L., Schäffer A.A., Zhang J., Zhang Z., Miller W., Lipman D.J. (1997). Gapped BLAST and PSI-BLAST: A new generation of protein database search programs. Nucleic Acids Res..

[32] Söding J., Biegert A., Lupas A.N. (2005). The HHpred interactive server for protein homology detection and structure prediction. Nucleic Acids Res..

[33] Larpin Y., Oechslin F., Moreillon P., Resch G., Entenza J.M., Mancini S. (2018). In vitro characterization of PlyE146, a novel phage lysin that targets Gram-negative bacteria. PLoS ONE.

[34] Lai M.J., Lin N.T., Hu A., Soo P.-C., Chen L.-H., Chang K.C. (2011). Antibacterial activity of *Acinetobacter baumannii* phage phiaB2 endolysin (LysAB2) against both Gram-positive and Gram-negative bacteria. Appl. Microbiol. Biotechnol..

[35] Huang G., Shen X., Gong Y., Dong Z., Zhao X., Shen W., Wang J., Hu F., Peng Y. (2014). Antibacterial properties of *Acinetobacter baumanniiphage* Abp1 endolysin (PlyAB1). BMC Infect. Dis..

[36] Rao G.R., Burma D.P. (1971). Purification and properties of phage P22-induced lysozyme. J. Biol. Chem..

[37] Miroshnikov K.A., Faizullina N.M., Sykilinda N.N., Mesyanzhinov V.V. (2006). Properties of the endolytic transglycosylase encoded by gene 144 of *Pseudomonas aeruginosa* bacteriophage phiKZ. Biochemistry.

[38] Sun Q., Kuty G.F., Arockiasamy A., Xu M., Young R., Sacchettini J.C. (2009). Regulation of a muralytic enzyme by dynamic membrane topology—Supplementary information. Nat. Struct. Mol. Biol..

[39] Vollmer W. (2008). Structural variation in the glycan strands of bacterial peptidoglycan. FEMS Microbiol. Reviews.

[40] Legotsky S.A., Vlasova K.Y., Priyma A.D., Shneider M.M., Pugachev V.G., Totmenina O.D., Kabanov A.V., Miroshnikov K.A., Klyachko N.L. (2014). Peptidoglycan degrading activity of the broad-range Salmonella bacteriophage S-394 recombinant endolysin. Biochimie.

[41] Oliveira H., Melo L.D.R., Santos S.B., Nóbrega F.L., Ferreira E.C., Cerca N., Azeredo J., Kluskens L.D. (2013). Molecular aspects and comparative genomics of bacteriophage endolysins. J. Virol..

