# Supplementary material for: Structure of an Acinetobacter Broad-Range Prophage Endolysin Reveals a C-Terminal α-Helix with the Proposed Role in Activity against Live Bacterial Cells
Source: Viruses. 2018 Jun 6;10(6):309. doi: 10.3390/v10060309 (PMC6024848; doi:10.3390/v10060309)
Supplement: Supplementary file 1 [file viruses-10-00309-s001.pdf]

Figure S1 Removal of the growth media components enhances the effect of Aclys on live bacterial cells

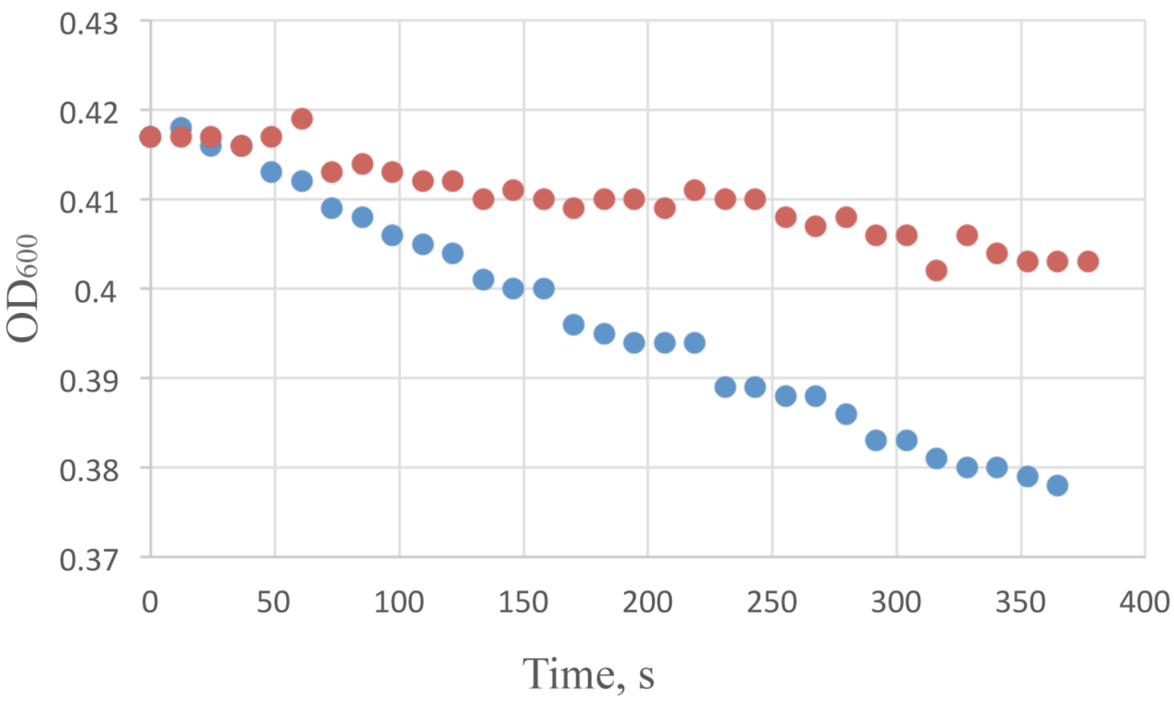

Figure S1. Bactericidal activity of AcLys (100◎g/mL) on viable *E. coli* CR63 cells washed with deionized water and resuspended in 20 mM Bis-Tris buffer pH 6.0 at 25 $_{o}$C (red). Decrease of *E. coli* CR63 optical density at the same enzyme concentration, but in LB media is shown in red.

Figure S2 Temperature dependence of AcLys activity

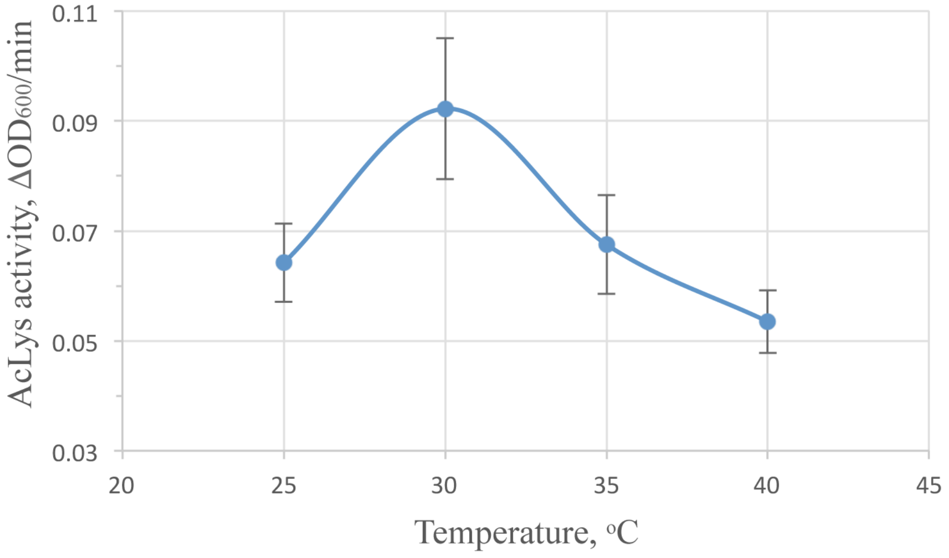

Figure S2. Temperature dependence of AcLys activity. The substrate - *E. coli* CR63 preliminary washed with deionized water and frozen at -70 ^{o}C. Reaction conditions 20 mM Bis-Tris (pH 6.0) AcLys 1 ◎g/mL. Activity was measured at the temperatures shown.

Figure S3. Dependence of AcLys activity on salt concentration

(A)

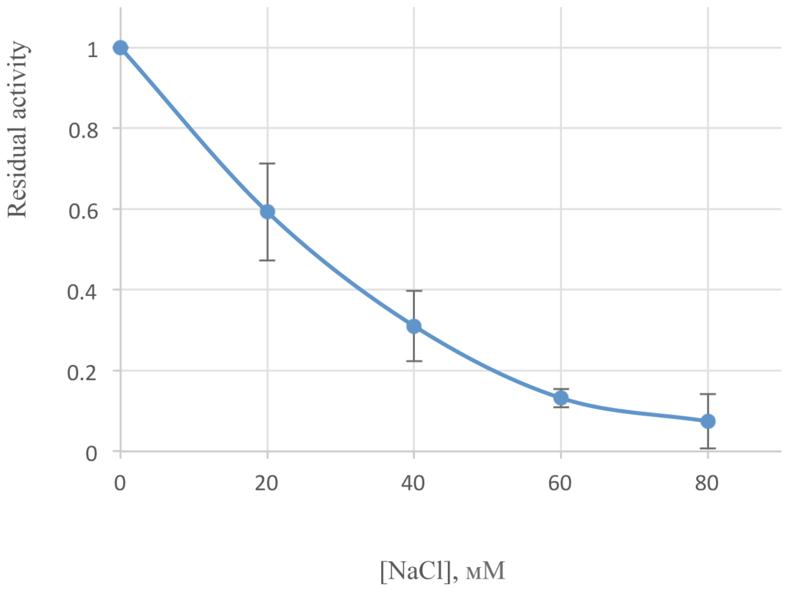

(B)

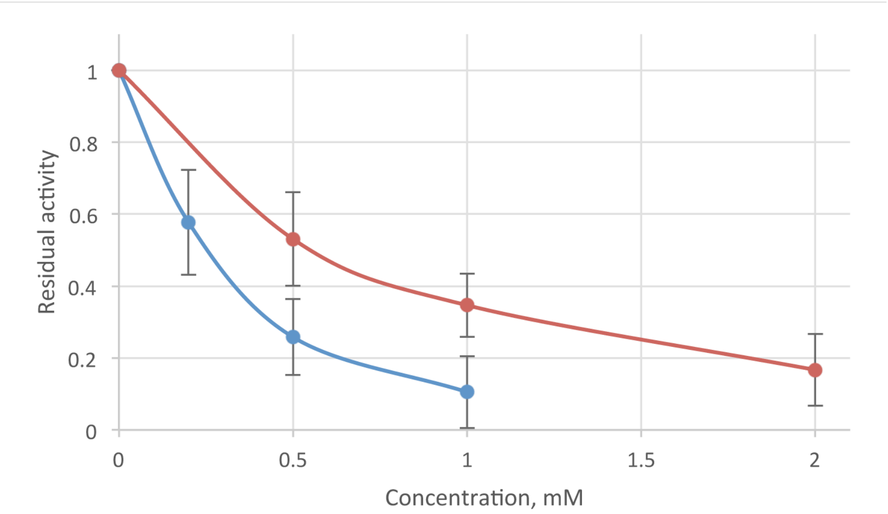

Figure S3. Dependence of AcLys activity on salt concentration. The substrate - *E. coli* CR63 preliminary washed with deionized water and frozen at -70 °C. AcLys concentration 1⊚g/mL. Reaction run at 25 °C. A) Influence of NaCl. Reaction conditions 20 mM Bis-Tris (pH 6.0) supplied with indicated concentrations of NaCl; B) Influence of divalent cations. Reaction conditions 20 mM Bis-Tris (pH 6.0) supplied with indicated concentrations of $MgCl_2$ (red) and $CaCl_2$ (blue).
